# Supplementary material for: Agricultural fungicides inadvertently influence the fitness of Colorado potato beetles, Leptinotarsa decemlineata, and their susceptibility to insecticides
Source: Sci Rep. 2018 Sep 5;8:13282. doi: 10.1038/s41598-018-31663-4 (PMC6125405; doi:10.1038/s41598-018-31663-4)

Supplementary Information: Agricultural fungicides inadvertently influence the fitness of Colorado potato beetles*, Leptinotarsa decemlineata,* and their susceptibility to insecticides

Justin Clements^1^, Sean Schoville^1^, Anna Clements^2^, Dries Amezian^1^, Tabatha Davis^1^, Benjamin Sanchez-Sedillo^1^, Christopher Bradfield^3^, Anders S. Huseth^4^, and Russell L. Groves^1^

1: Department of Entomology, University of Wisconsin-Madison, Madison, WI 53706, USA

2: Department of Orthopedics and Rehabilitation, University of Wisconsin-Madison, Madison, WI 53706, USA

3: Department of Oncology, University of Wisconsin-Madison, Madison, WI 53706, USA

4: Department of Entomology and Plant Pathology, North Carolina State University, Raleigh, NC 27695, USA

Supplementary Table S1. Quantitative PCR primers, primer efficiency, and transcript identification including associated BLASTx NCBI accession number referenced by Clements et al. ^8,9,27^.

|  | **Forward Primer (5’-3’)** | **Reverse Primer (5’-3’)** | **Primer Efficiency** | **Transcript BLAST x Result** | **NCBI Accession Numbers** |
| --- | --- | --- | --- | --- | --- |
| RP4  (Reference) | AAAGAAACGAGCATTGCCCTTCCG | TTGTCGCTGACACTGTAGGGTTGA | 1.93 |  |  |
| LDEC003961  (Cuticular protein) | ACCTGCTGCCGGTATTATTG | TACAGTTCCAGAGGGTCCAG | 1.96 | Cuticular protein | XP_966639.1 |
| LDEC016769  (Cytochrome p450) | CAGGTCTGACAAGGATATGGTTAG | TCCAGAGCTTTCGGATGATTC | 1.98 | Cytochrome p450 | XP_973153.1 |
| comp103658  (Cytochrome p450) | TCCTCACTGAATCTTTCTGGATCG | AGCCCAGATGAGAAGCCATTAC | 2.02 | Cytochrome p450 9z4 | NP_001164248 |
| comp111691  (Cytochrome p450) | TGCCGTCTCCTAGCTTTGTAAC | CGGATTCGATACCAGTTCAACAC | 2.05 | Cytochrome p450 monooxygenase | XP_972348 |
| comp114026 (Glutathione synthetase) | CAGAGCAGGGTATGAACCTAATC | CCAGCCAAGTGATACTGAATCG | 1.97 | Glutathione synthetase | XP_968070 |
| comp115309 (Cytochrome P450) | CGAGAAATGCGACCTATTCTCAG | ACACAGTCTTGGTCTTTCTTGAG | 1.98 | Cytochrome  P450  9Z26 | KJ476503.1 |

Supplementary Table S2. Mean estimates of fungicide and insecticide inputs by state.

| **State** | **Mean insecticide application number** | **Mean fungicide applications** | **Mean pounds neonicotinoid applied (±SE)** | **Mean pounds fungicide applied (±SE)** |
| --- | --- | --- | --- | --- |
| Maine | 1.2 | 7.69 | 0.153 (0.018) | 5.288 (0.375) |
| Michigan | 1.72 | 6.8 | 0.223 (0.033) | 5.824 (0.703) |
| Wisconsin | 1.89 | 7.59 | 0.276 (0.024) | 6.822 (0.441) |
| Minnesota | 1.71 | 5.58 | 0.206 (0.028) | 5.313 (0.876) |
| North Dakota | 1.86 | 6.24 | 0.128 (0.034) | 5.859 (0.633) |
| Colorado | 1.53 | 2.1 | 0.078 (0.033) | 1.536 (0.171) |
| Idaho | 1.71 | 2.27 | 0.196 (0.023) | 1.849 (0.14) |
| Washington | 1.9 | 2.94 | 0.181 (0.027) | 2.64 (0.189) |

Supplementary Figure S1. Percent survivorship of second instar *Leptinotarsa decemlineata* larva exposed to chronic doses of field relevant rates of chlorothalonil, boscalid, or control.


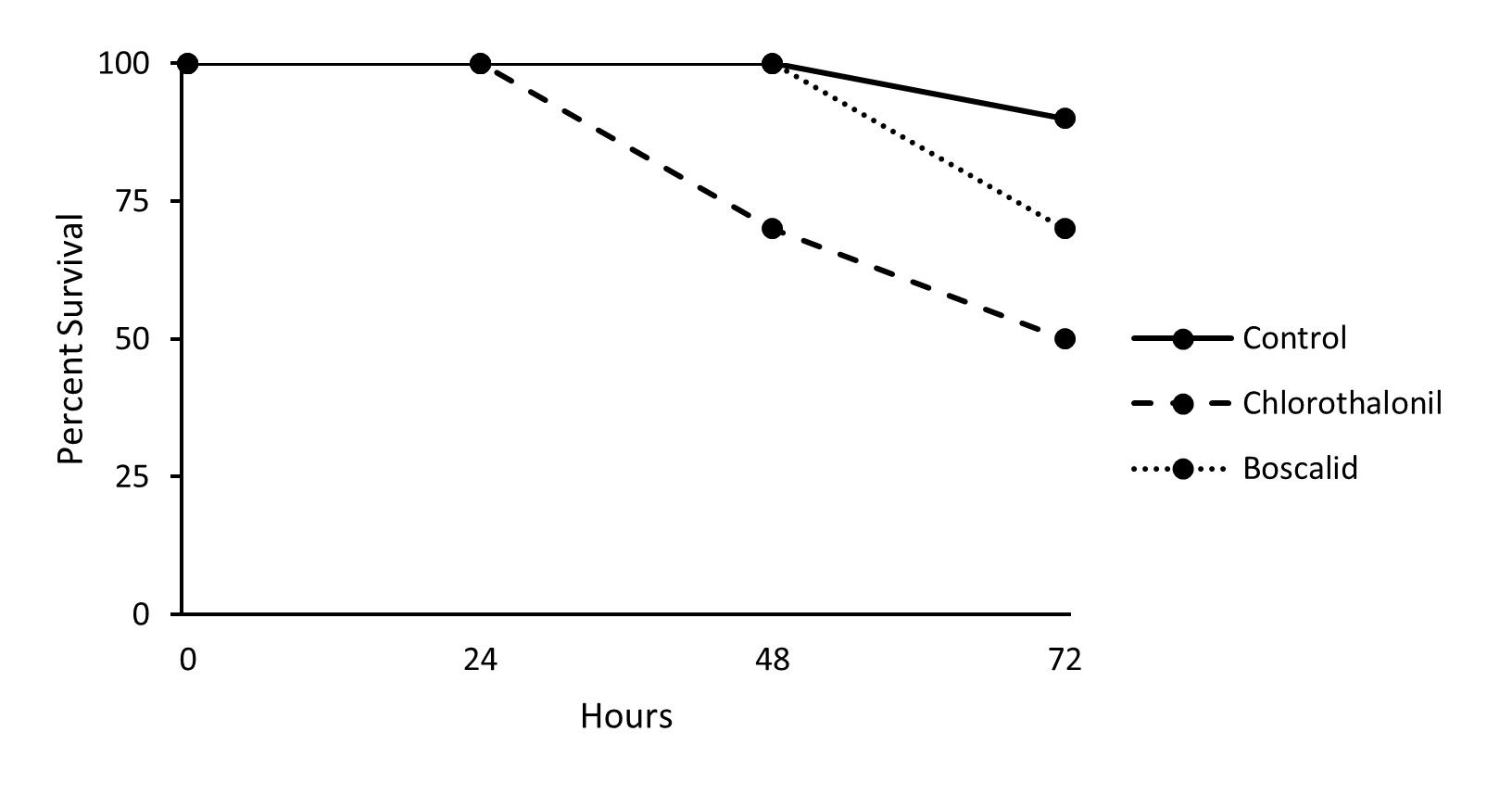

Supplement: Supplementary file 1 — Supplementary Information [file 41598_2018_31663_MOESM1_ESM.docx]
